# Radiation-induced parotid changes in oropharyngeal cancer patients: the role of early functional imaging and patient−/treatment-related factors

**DOI:** 10.1186/s13014-018-1137-4

**Published:** 2018-10-01

**Authors:** Simona Marzi, Alessia Farneti, Antonello Vidiri, Francesca Di Giuliano, Laura Marucci, Filomena Spasiano, Irene Terrenato, Giuseppe Sanguineti

**Affiliations:** 1 Medical Physics Laboratory, IRCCS Regina Elena National Cancer Institute, Via Elio Chianesi 53, 00144 Rome, Italy; 2 Department of Radiotherapy, IRCCS Regina Elena National Cancer Institute, Via Elio Chianesi 53, 00144 Rome, Italy; 3 Radiology and Diagnostic Imaging Department, IRCCS Regina Elena National Cancer Institute, Via Elio Chianesi 53, 00144 Rome, Italy; 4 Department of Biomedicine and Prevention, University of Rome “Tor Vergata”, Viale Oxford 81, 00133 Rome, Italy; 5 Biostatistics-Scientific Direction, IRCCS Regina Elena National Cancer Institute, Via Elio Chianesi 53, 00144 Rome, Italy

**Keywords:** Diffusion magnetic resonance imaging, Perfusion magnetic resonance imaging, Radiotherapy, Parotid gland, Oropharyngeal Cancer

## Abstract

**Background:**

Functional magnetic resonance imaging may provide several quantitative indices strictly related to distinctive tissue signatures with radiobiological relevance, such as tissue cellular density and vascular perfusion. The role of Intravoxel Incoherent Motion Diffusion Weighted Imaging (IVIM-DWI) and Dynamic Contrast-Enhanced (DCE) MRI in detecting/predicting radiation-induced volumetric changes of parotids both during and shortly after (chemo)radiotherapy of oropharyngeal squamous cell carcinoma (SCC) was explored.

**Methods:**

Patients with locally advanced oropharyngeal SCC were accrued within a prospective study offering both IVIM-DWI and DCE-MRI at baseline; IVIM-DWI was repeated at the 10th fraction of treatment. Apparent diffusion coefficient (ADC), tissue diffusion coefficient D_t_, perfusion fraction *f* and perfusion-related diffusion coefficient D^*^ were estimated both at baseline and during RT. Semi-quantitative and quantitative parameters, including the transfer constant K^trans^, were calculated from DCE-MRI. Parotids were contoured on T2-weighted images at baseline, 10th fraction and 8th weeks after treatment end and the percent change of parotid volume between baseline/10th fr (∆Vol_10fr_) and baseline/8th wk. (∆Vol_post_) computed.

Correlations among volumetric changes and patient-, treatment- and imaging-related features were investigated at univariate analysis (Spearman’s Rho).

**Results:**

Eighty parotids (40 patients) were analyzed. Percent changes were 18.2 ± 10.7% and 31.3 ± 15.8% for ∆Vol_10fr_ and ∆Vol_post_, respectively. Among baseline characteristics, ∆Vol_10fr_ was correlated to body mass index, patient weight as well as the initial parotid volume. A weak correlation was present between parotid shrinkage after the first 2 weeks of treatment and dosimetric variables, while no association was found after radiotherapy. Percent changes of both ADC and D_t_ at the 10th fraction were also correlated to ∆Vol_10fr_. Significant relationships were found between ∆Vol_post_ and baseline DCE-MRI parameters.

**Conclusions:**

Both IVIM-DWI and DCE-MRI can help to detect/predict early (during treatment) and shortly after treatment completion the parotid shrinkage. They may contribute to clarify the correlations between volumetric changes of parotid glands and patient−/treatment-related variables by assessing individual microcapillary perfusion and tissue diffusivity.

**Electronic supplementary material:**

The online version of this article (10.1186/s13014-018-1137-4) contains supplementary material, which is available to authorized users.

## Background

Radiotherapy is involved in the treatment of the majority of patients with non-metastatic head and neck squamous cell carcinoma [1]. Despite intensity modulated radiotherapy (IMRT), xerostomia is still a frequent radiation-induced complication with implications on patients quality of life [2, 3]. More importantly, there is some degree of inconsistency between dosimetric data of the parotids at planning and long-term patient reported outcomes [4]. Among possible explanations, variations in intrinsic radiosensitivity among individuals [5], discrepancies between objective outcome and patient perception [6] and variations in parotid gland (PG) dosage during the course of IMRT due to shrinkage have been advocated [7, 8].

Thought the pathophysiology of parotid shrinkage during radiotherapy remains unclear [9], image-based scoring of toxicity based on early changes of irradiated organs can provide a timely, objective and consistent endpoint for patient counseling and treatment strategy adaptation [5, 10–12]. In this regard, functional magnetic resonance imaging (MRI) techniques, such as diffusion- and perfusion- MRI, may have an important role, as they provide several quantitative indices strictly related to distinctive tissue signatures with radiobiological relevance, such as tissue cellular density and vascular perfusion [12, 13].

Dynamic contrast-enhanced (DCE) MRI is a well-established technique to evaluate the hemodynamic properties of several tissues, such as vessel leakiness and permeability, by means of serial MRI scans taken before and after the intravenous injection of a contrast agent [13, 14]. Moreover, diffusion-weighted imaging (DWI) provides insights into cellular architecture, being sensitive to the thermally driven motion of water molecules in tissues [15]. Therefore, DWI indirectly assesses cell depopulation due to radiation-induced changes. Intravoxel Incoherent Motion (IVIM) imaging is a further evolution of DWI potentially able to provide both diffusion and perfusion imaging results with the additional advantage of being completely non-invasive, as it does not require contrast injection [16, 17]. IVIM-DWI assumes that the microcirculation of blood can be thought as a *pseudo-diffusion* incoherent motion, because the capillary network does not have a specific spatial orientation within a single image voxel. To separate the perfusion effects from those of thermal diffusion, IVIM-DWI requires the acquisition of images with multiple b value, where b is a factor dependent on the gradient pulse sequence (i.e. pulse duration and strength of the diffusion gradient). The perfusion effect on the signal attenuation may be significant at low b values (0–150 s/mm2) in well-vascularized tissues, while it is assumed to vanish at higher b values due to the larger drop in signal compared to the thermal diffusivity effect. By using a bi-exponential function to model the IVIM signal intensity at increasing b values [16], both perfusion-related and perfusion-free diffusion parameters may be derived, as described below in more detail.

In January 2016 we started a prospective study funded by the Italian Association for Cancer Research (AIRC, project No.17028) in locally advanced oropharyngeal SCC investigating the ability of both DCE-MRI and IVIM-DWI to predict tumor response to (chemo-)IMRT. As part of this ongoing study, we investigated also volumetric changes of parotid glands during and shortly after treatment completion. Here we report the results on 40 consecutive patients.

## Methods

### Patient population& treatment

To be eligible, patients had to fulfill all the following criteria: (i) age older than 18 years; (ii) Karnofsky performance status > 80; (iii) pathologically confirmed squamous cell carcinoma of the oropharynx; (iv) stage III or IV without distant metastases according to the 7th edition of the AJCC Cancer Staging Manual; (v) treatment with radiotherapy + chemotherapy. Exclusion criteria included: any contraindication for MR examination; prior surgery, CHT (including induction chemotherapy) or radiotherapy to the primary disease and the neck. Moreover, specific informed consent was obtained from each patient before enrolling. The study protocol was approved by the local Institutional Review Board.

All patients were offered IMRT and concomitant cisplatin (CDDP), either 100 mg/m^2^ for three cycles every 21 days or weekly 40 mg/m^2^ for 6 cycles. If patients were not medically fit, IMRT alone was delivered. A simultaneous integrated boost technique delivering the following total doses in 35 fractions was used: 70 Gy to macroscopic disease; 63 Gy to regions at high risk of microscopic disease; 58.1 Gy to regions at intermediate risk of microscopic disease [18]. A 7-field approach with 6 MV photons was used. For each patient, the planned mean dose to the single parotid gland, D_mean_(Gy), and the percentage of parotid volume receiving ≥30 Gy, V_30_(%), were calculated from the treatment planning system (Eclipse version 13.5.37, Varian Medical System, Palo Alto, CA) and recorded.

### MR imaging protocols

MRI scans were performed with a 1.5-T system (Optima MR 450w, GE Health-care, Milwaukee, Wisconsin) with dedicated 16-channels receive-only RF coils: a head coil, a surface neck and a spine coil. In addition to standard imaging, all patients underwent three serial MRI examinations: before treatment, after the 10th fraction of chemoradiotherapy and 8 weeks after the end of treatment. MRI scanning was avoided on the same day of chemotherapy administration.

At baseline, the MRI acquisition protocol included both IVIM-DWI and DCE-MRI examinations. However, only IVIM-DWI was repeated at the 10th fraction to reduce the use of intravenous contrast agent. Both coronal and axial fast spin echo T_2_-weighted images (acquisition matrix, 288 × 256; field of view, 26–28 cm; TR/TE, 5739/100 ms; slice thickness, 4 mm; inter-slice gap, 5; acquisition time, 2 min and 40 s) were acquired in all MRI exams.

IVIM-DWI was performed using single-shot echo-planar imaging with the following parameters: acquisition matrix 128 × 128, field of view 26–28 cm, TR/TE 4500 ms/77 ms, number of slices 30–33, slice thickness 4 mm, and intersection gap 1 mm. Nine different *b* values (*b* = 0, 25, 50, 75, 100, 150, 300, 500, and 800 s/mm^2^) were used, with all diffusion-sensitizing gradients applied in three orthogonal directions to obtain trace-weighted images.

To enable the perfusion-sensitive information to be accurately extracted from DWI, most of the b values (six) were chosen within the lower range 0–150 s/mm^2^, while three b values in the range 300–800 s/mm^2^ were considered adequate to estimate the perfusion-free tissue diffusion coefficient. The maximum b value was limited to 800 s/mm^2^ as it usually provides a sufficient signal to noise ratio and a good lesion cospicuity in the head and neck region at 1.5-T.

Three signal averages were chosen for *b* values ranging from 0 to 300 s/mm^2^, four for a *b* value of 500 s/mm^2^, and five for a *b* value of 800 s/mm^2^. Array Spatial Sensitivity Encoding Technique imaging, with a scan-time reduction factor of 2, was used, with a resulting scanning time of 6 min and 13 s.

DCE-MRI involved a 3D fast-spoiled gradient echo sequence with the following characteristics: TR/TE:3.82/1.18 ms; flip angle: 30°; acquisition matrix: 128 × 128; field of view: 28 cm; number of slices: 32; slice thickness: 4 mm; spacing between slices: 2 mm.

Sixty dynamic volumes were acquired consecutively, with a temporal resolution of 5 s and a total scanning time of 5 min. At the fourth dynamic volume, 0.1 mmol/kg body weight of gadopentetatedimeglumine contrast agent was administered intravenously, at a rate of 3 ml/s.

### Parotid gland delineation

For each patient, a single observer manually outlined the entire PG on T2-weighted images acquired at each time point. 3D Slicer Software (version 4.6.2) was used for image visualization and for gland segmentation [19]. Vascular structures were meticulously excluded from the delineated PG volume. These contours were propagated onto DCE-MRI images using the 3D Slicer module *Expert Automated Registration*.

**Fig. 1:**
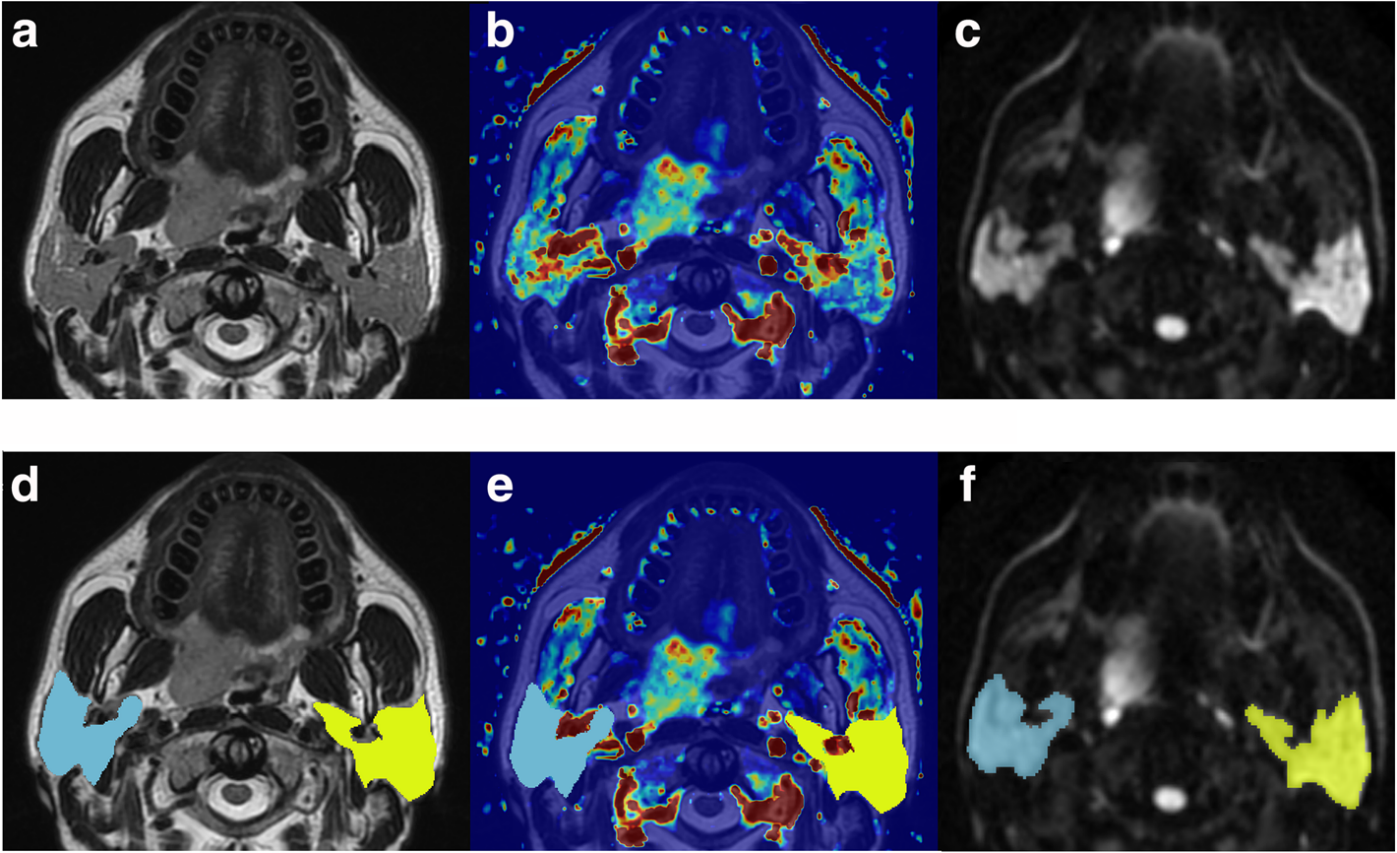
Central section of parotids in a 44-year-old man affected by a tonsil carcinoma on the axial T2-weighted image (**a**), on the map of K^trans^ (**b**) and on the diffusion-weighted image obtained with b of 800 s/mm^2^ (**c**) in the same anatomic location. Bottom: user-defined parotid contours overlaid on the axial T2-weighted image (**d**) and on the corresponding map of K^trans^ (**e**) and diffusion-weighted image obtained with b of 800 s/mm^2^ (**f**)

The parotid contours obtained on T2-weighted images also served as a guide to parotid gland delineation on DWI sequences with b = 800 s/mm^2^. An example of parotid delineation is illustrated in Fig. 1.

### Calculation of IVIM-diffusion parameters

Dedicated scripts were developed in Matlab code (Release 7.10.0, The Mathworks Inc., Natick, Massachusetts) for quantitative image analyses. The median value of the signal from all voxels within the entire gland was derived for each b value and its variation at increasing b was modeled using the following bi-exponential function by a nonlinear constrained minimization algorithm [16]: $$ \raisebox{1ex}{${\mathrm{S}}_{\mathrm{b}}$}\!\left/ \!\raisebox{-1ex}{${\mathrm{S}}_0$}\right.=\left(1-\mathrm{f}\right)\bullet {\mathrm{e}}^{-\mathrm{b}\bullet \mathrm{D}}+\mathrm{f}\bullet {\mathrm{e}}^{-\mathrm{b}\bullet \left(\mathrm{D}+{\mathrm{D}}^{\ast}\right)} $$ where *S*_*b*_ is the signal intensity with diffusion weighting *b*, *S*_*0*_ is the signal intensity for a *b* value of 0 s/mm^2^, *f* is the fractional volume of capillary blood*,* D_t_ is the tissue diffusion coefficient (in mm^2^/s), and D* is the perfusion-related diffusion coefficient (in mm^2^/s).

As described elsewhere [20], a ‘two step’ fitting method was implemented to avoid overfitting: firstly the D_*t*_ value was derived from data at *b* values of 300, 500 and 800 s/mm^2^ using a mono-exponential fit; secondly fixing D_*t*_ at the value estimated above, S0, D*, and *f* were determined according to Eq.1. To avoid the risk of suboptimal solutions, one hundred simultaneous optimization processes were launched for each fit, starting from randomly chosen initial values of parameters between fixed bounds. The residual sum of squares value of each optimization process was recorded and the minimum value was used to assess the best solution for the parameters.

The conventional ADC was also derived from data at *b* values of 0, 500 and 800 s/mm^2^, using a mono-exponential model [15].

### Calculation of the DCE-MRI parameters

The DCE-MRI data were analyzed using a commercial software package (GenIQ General, GE Advanced Workstation, Palo Alto, CA). The pharmacokinetic analysis was performed based on the Toft model: the quantitative parameters K^trans^, the transfer constant between plasma and the extravascular extracellular space (EES), K_ep_, the transfer constant between EES and plasma and v_e_, the fractional volume of EES were calculated [14]. A semi-quantitative parameter, the initial area under gadolinium concentration curve or IAUGC (calculated from the bolus arrival to the first 90 s) was also derived. The median of each parameter, computed from the distribution of the voxel-level values within the entire gland, was used for statistical purposes.

### Statistics

The change in parotid volume was estimated as the percent volume reduction at both the 10th fraction of RT (∆Vol_10fr_) and the 8th week after the end of IMRT (∆Vol_post_).

The Saphiro-Wilk test was used to test the normality of the data. As some of the variables (about 49%) were not normally distributed, non-parametric analyses were performed, as they are based on distribution-free methods. The Wilcoxon test for paired samples was used to assess whether changes over time were significant. Correlations among volumetric changes at the time points and various patient-, treatment- and imaging-related features were investigated at univariate analysis with the Spearman test. We did not apply any adjustment to the *p*-values for multiple comparisons because we have only tested multiple variables within a single group and not between groups. A p level < 0.05 was considered to indicate statistical significance. SPSS software (SPSS version 21, SPSS Inc., Chicago, IL, USA) was used for the statistical analyses.

## Results

Forty patients/80 parotids were accrued and analyzed. Selected patient and tumor characteristics are summarized in Table 1. All patients received the planned radiotherapy dose and all but 7 patients received concomitant chemotherapy as well. The average (±SD) patient weight loss at the 10th fraction and at the end of RT were 2.0 ± 2.7 Kg (− 2.7% ± 3.8%) and 6.8 ± 4.4 Kg (9.0% ± 5.1%), respectively. At the 10th fraction, the parotid volume decreased from a mean (±SD) value of 32.3 (±9.4) cm^3^ to 26.7 (±9.0) cm^3^, with a mean ∆Vol_10fr_ of 18.2% (±10.7%). Eight weeks after RT, it decreased to 22.7 (±8.9) cm^3^, with a mean ∆Vol_post_ of 31.3% (±15.8); all but two patients were not evaluated at this time point as they died of progressive disease. No correlation was found between ∆Vol_10fr_/∆Vol_post_ and the patient weight loss at the 10th fraction and at the end of RT.

**Table 1:** Selected patient and tumor characteristics

Characteristic	Parameter
Patient number	40 (80 parotids)
Sex (M/F)	30/10
Age (years)	
Mean (range)	65.5 (46–81)
Primary tumor site	
Tonsil	26 (65%)
Base of Tongue	14 (35%)
T stage	
T1	4 (10%)
T2	11 (27.5%)
T3	7 (17.5%)
T4	15 (37.5%)
T4a	3 (7.5%)
N stage	
N0	4 (10%)
N1	4 (10%)
N2a	2 (5%)
N2b	15 (37.5%)
N2c	14 (35%)
N3	1 (2.5%)
Parotid Radiation Dose	
D_mean_ (Gy)	35.8 ± 8.9
V_30_(%)	53.6 ± 19.9

Abbreviations: *D*_*mean*_ Planned mean dose to the parotid gland, *V*_*30*_*(%)* Percentage of parotid volume receiving a dose ≥30 Gy

At baseline, the average (±SD) values for K^trans^, K_ep_, v_e_ and IAUGC were 0.546 (±0.290) min^− 1^, 2.728 (±1.146) min^− 1^, 0.178 (± 0.061) (fractional units) and 0.287 (±0.095) (arbitrary units), respectively. Summary statistics of the diffusion parameters and their variations during treatment are shown in Table 2. Only the changes of ADC and D_t_ over time were significant (*p* < 0.001 for both).

**Table 2:** Summary statistics of the diffusion parameters and their variations during treatment

Parameter^a^	before RT	at the 10th	Variation from baseline (%)	*p*
ADC (10^−3^ mm^2^/s)	1.275 ± 0.158	1.434 ± 0.205	13.6 ± 18.2	**< 0.0001**
D_t_ (10^−3^ mm^2^/s)	0.949 ± 0.153	1.072 ± 0.184	14.5 ± 20.8	**< 0.0001**
*f*(%)	15.8 ± 4.7	16.3 ± 5.1	8.8 ± 41.1	0.420
D* (10^−3^ mm^2^/s)	28.4 ± 18.1	31.7 ± 2.1	57.9 ± 156.5	0.185
D* × *f* (10^−3^ mm^2^/s)	424 ± 305	466 ± 227	51.0 ± 129.1	0.076

^a^Average ± standard deviation. P values refer to Wilcoxon test. Statistically significant *p*-values are bold. Abbreviations: *ADC* Apparent diffusion coefficient, *D*_*t*_ Tissue diffusion coefficient, D* Perfusion-related diffusion coefficient, *f(%)* Perfusion fraction, D* *× f* Product of D* by *f*

### Correlation between parotid shrinkage and variables at baseline

**Fig. 2:**
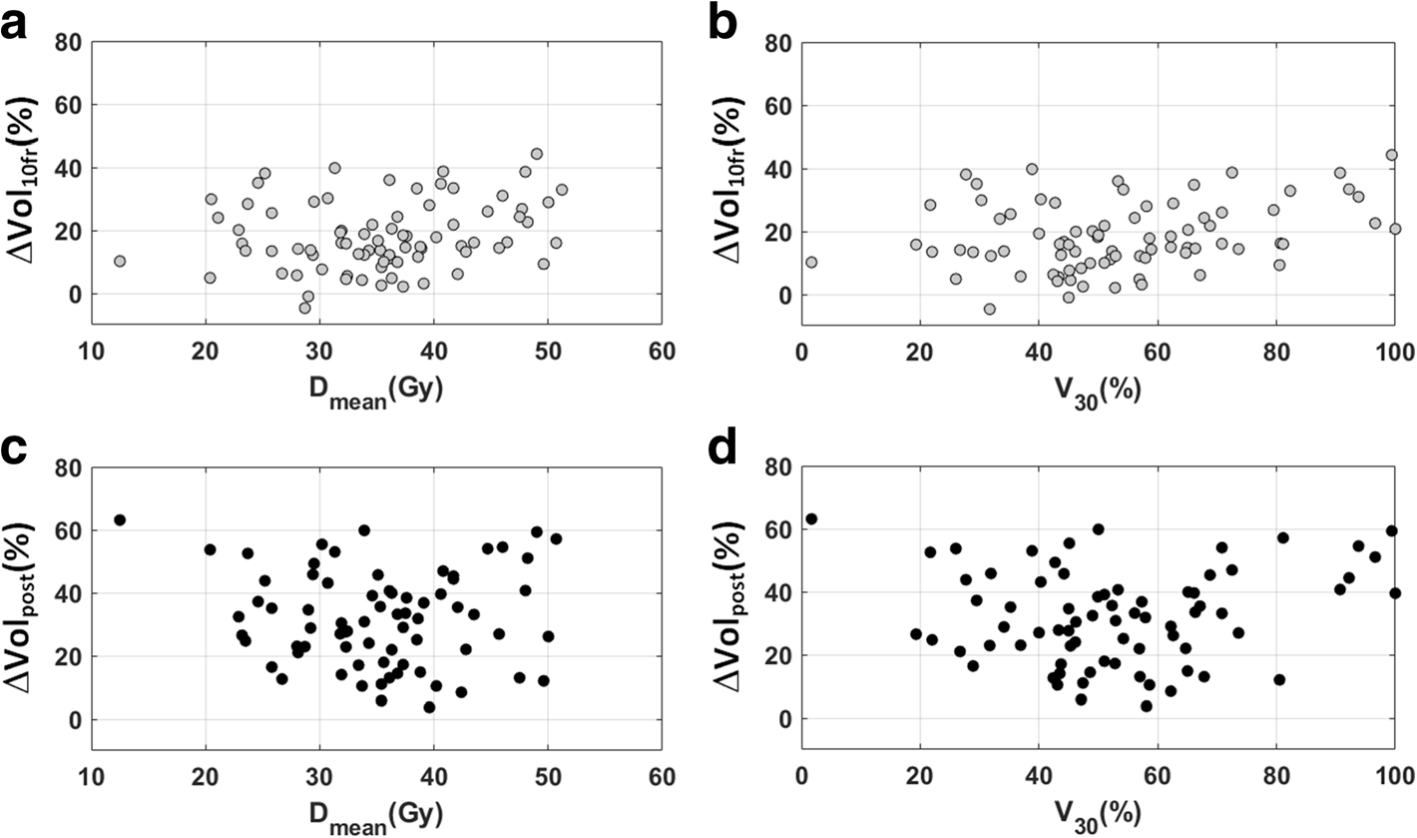
Scatter plots of parotid shrinkage(%) at the 10th fraction (∆Vol_10fr_) versus planned mean dose to the parotid gland D_mean_ (**a**) and percentage of parotid volume receiving a dose ≥30 Gy, V_30_(%) (**b**). Analogously, scatter plots of parotid shrinkage(%) 8 weeks after RT (∆Vol_post_) versus D_mean_ (**c**) and V_30_(%) (**d**)

Table 3 reports the relationships between percent parotid shrinkage and various covariates at baseline. A significant correlation was found between ∆Vol_10fr_ and initial parotid volume, patient weight, BMI, D_mean_ and V_30_. Scatter plots of parotid shrinkage versus D_mean_ and V_30_(%) are depicted in Fig. 2. Among image-related parameters, only K_ep_ was weakly associated to parotid shrinkage (Rho = 0.239, *p* = 0.038) while K^trans^ showed only a trend towards statistical significance (Rho = 0.210, *p* = 0.069).

**Table 3:** Results of Spearman’s correlation tests between parotid shrinkage and imaging/ anthropometric parameters at baseline

Variable at baseline	∆Vol_10fr_(%)		∆Vol_post_(%)	
	Spearman’s Rho	*p*-value	Spearman’s Rho	*p*-value
ADC	− 0.185	0.100	−0.010	0.932
D_*t*_	−0.099	0.385	−0.061	0.609
*f*	−0.056	0.620	−0.002	0.986
D*	−0.019	0.865	−0.188	0.109
D* × *f*	−0.007	0.950	−0.167	0.154
K^trans^	0.210	0.069	0.434	**< 0.001**
K_ep_	0.239	**0.038**	0.349	**0.003**
v_e_	0.025	0.832	0.174	0.143
IAUGC	0.139	0.231	0.375	**0.001**
Parotid volume	−0.242	**0.031**	−0.056	0.636
Age	−0.108	0.341	0.045	0.702
Weight	−0.396	**< 0.001**	−0.135	0.253
BMI	−0.368	**0.001**	−0.127	0.281
D_mean_(Gy)	0.226	**0.044**	−0.007	0.951
V_30_(%)	0.266	**0.017**	0.086	0.468

Statistically significant p-values are bold. Abbreviations: *∆Vol*_*10fr*_ Parotid shrinkage(%) at the 10th fraction, *∆Vol*_*post*_ Parotid shrinkage(%) 8 weeks after RT, *ADC* Apparent diffusion coefficient, *D*_*t*_ Tissue diffusion coefficient, D* Perfusion-related diffusion coefficient, *f(%)* Perfusion fraction, D* × f Product of D* by *f*, *∆ADC* ADC variation (%) relative to the pretreatment value (analogously for the other imaging variables), *K*^*trans*^ Transfer constant between plasma and EES (extravascular extracellular space), *K*_*ep*_ Transfer constant between EES and plasma, *v*_*e*_ Fractional volume of EES, *IAUGC* Initial area under gadolinium concentration curve, *BMI* Body mass index, *D*_*mean*_ Planned mean dose to the parotid gland, *V*_*30*_*(%)* Percentage of parotid volume receiving a dose ≥30 Gy

Interestingly, none of the selected patient- and treatment-related variables was correlated to percent shrinkage at 8 weeks after treatment completion while several DCE-MRI-related parameters showed a moderate correlation at this time point, with stronger evidence (smaller significant *p* values) than at the 10th fraction (Table 3).

### Correlation between parotid shrinkage and diffusion parameters during treatment

The correlation between parotid shrinkage and DWI-IVIM parameters at the 10th fraction are reported in Table 4. ∆Vol_10fr_ significantly correlated with D_t_ and the percent change of both ADC and D_t_ between baseline and the 10th fraction.

**Table 4:** Results of Spearman’s correlation tests between parotid shrinkage and diffusion parameters during treatment

Variable at the 10th fraction	∆Vol_10fr_(%)		∆Vol_post_(%)	
	Spearman’s Rho	*p*-value	Spearman’s Rho	*p*-value
ADC	0.121	0.285	0.103	0.383
D_*t*_	0.286	**0.011**	0.289	**0.013**
*F*	−0.057	0.614	−0.232	**0.047**
D*	−0.114	0.314	0.122	0.299
D* × *f*	−0.153	0.176	−0.013	0.910
∆ADC	0.228	**0.042**	0.173	0.141
∆D_*t*_	0.319	**0.004**	0.372	**0.001**
∆*f*	0.022	0.844	−0.242	**0.038**
∆D*	−0.117	0.301	0.192	0.101
∆D* × *f*	−0.119	0.293	0.096	0.416

Statistically significant p-values are bold. Abbreviations: *∆Vol*_*10fr*_ Parotid shrinkage(%) at the 10th fraction, *∆Vol*_*post*_ Parotid shrinkage(%) 8 weeks after RT, *ADC* Apparent diffusion coefficient, *D*_*t*_ Tissue diffusion coefficient, D* Perfusion-related diffusion coefficient, *f(%)* Perfusion fraction, D* × f Product of D* by *f*, *∆ADC* ADC variation (%) relative to the pretreatment value (analogously for the other imaging variables)

A significant relationship was found between the final parotid shrinkage ∆Vol_post_ and D_t_, *f* and the changes in D_t_ and *f*. Moreover, ∆Vol_post_ was strongly related to ∆Vol_10fr_ (Rho = 0.471, *p* < 0.001).

Finally, changes in ADC/D_t_ were significantly related to D_mean_ (*p* = 0.001, *p* = 0.011, respectively) and V_30_(%) (p < 0.001, *p* = 0.001, respectively) (Additional file 1: Table S3).

### Illustrative cases

**Fig. 3:**
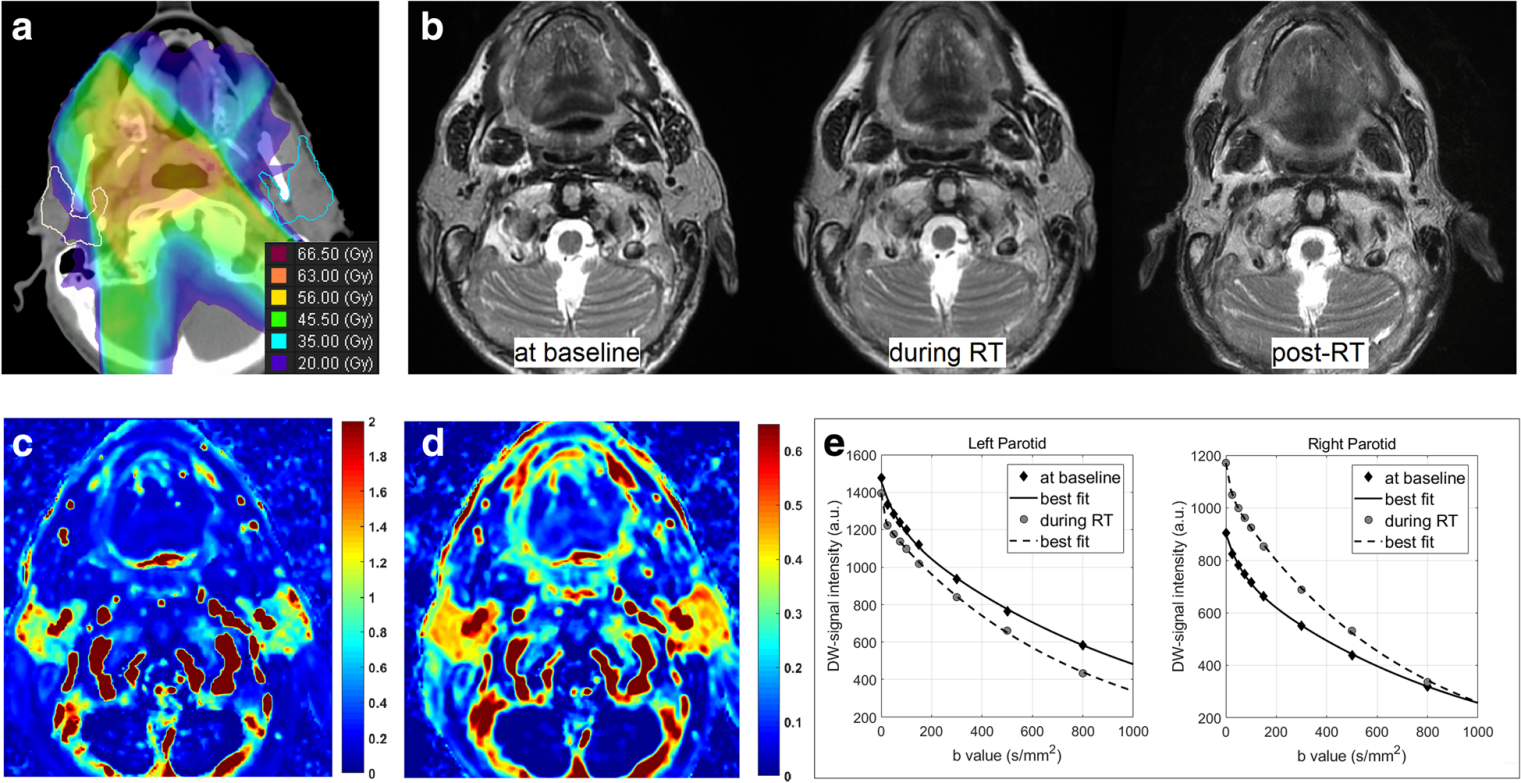
A 68-year-old man affected by a tonsil carcinoma: dose distribution overlaid on the central section of the parotid glands on axial CT (**a**); T2-weighted images before, at 2 weeks of radiotherapy and 8 weeks after radiotherapy (**b**) documenting a substantial gland shrinkage; the corresponding maps of K^trans^ in min^− 1^ (**c**) and IAUGC in a.u. (**d**), at baseline, indicating well perfused parotids*.* Curves of DW-signal attenuation of parotids at baseline and during RT (**e**): the modification of the DW-signal attenuation curves during RT documents a noticeable increase in tissue water diffusivity, suggesting a considerable decrease in cell density as a consequence of the radiation damage

**Fig. 4:**
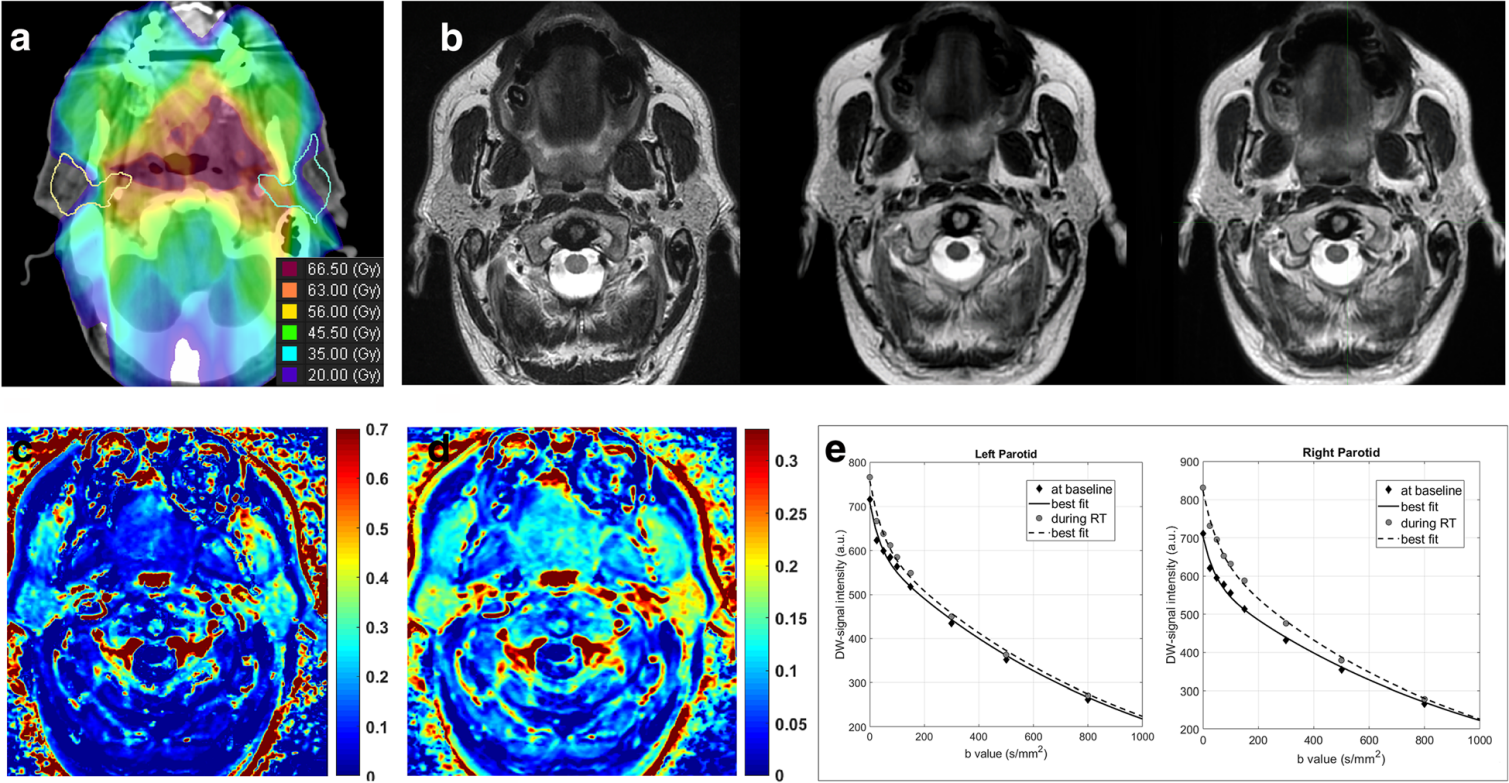
A 48-year-old man affected by a base of the tongue carcinoma: dose distribution overlaid on the central section of the parotid glands on axial CT (**a**); T2-weighted images before, at 2 weeks of radiotherapy and 8 weeks after radiotherapy (**b**) showing a small final parotid shrinkage; the corresponding maps of K^trans^ in min^− 1^ (**c**) and IAUGC in a.u. (**d**), at baseline, indicating moderately perfused glands. Correspondingly, a slight difference was observed between the rates at which the DW-signal attenuation of the parotids decreases during RT (**e**)

Two illustrative cases are presented in Figs. 3 and 4. In Fig. 3, the case of a 68-year-old man affected by a tonsil carcinoma is shown, whose initial weight and BMI were 65.5 Kg and 21.1 (Kg/m^2^), respectively. The three serial MR scans documented a substantial gland shrinkage, while the corresponding perfusion maps indicated well perfused parotids at baseline (the average K^trans^ and IAUGC of parotids were 0.868 min^− 1^ and 0.404, respectively). Curves of DW-signal attenuation of parotids at baseline and during RT demonstrated a noticeable increase in tissue water diffusivity, suggesting a considerable decrease in cell density as a consequence of the radiation damage (the average increase in ADC and D_t_ of parotids were 17% and 33%, respectively). In Fig. 4, the case of a 48-year-old man affected by a base of the tongue carcinoma is illustrated, whose initial weight and BMI were 97 Kg and 28.3 (Kg/m^2^), respectively. The three serial MR scans showed only a small final parotid shrinkage, while the perfusion maps indicated moderately perfused glands at baseline (the average K^trans^ and IAUGC of parotids were 0.276 min^− 1^ and 0.191, respectively). Correspondingly, a slight difference was observed between the rates at which the DW-signal attenuation of the parotids decreases during RT, suggesting a limited radiation-induced decrease in cell density (the average increase in ADC and D_t_ of parotids were 9% and 6%, respectively).

## Discussion

Image-based parameters evaluating early changes of irradiated organs have shown the potential to guide both treatment optimization and personalization, as supported by the increasing number of data published in the last few years [5, 21–26]. In the present study we investigated whether either IVIM-DWI or DCE-MRI can provide quantitative indices with radiobiological relevance, as tissue cellular density and vascular perfusion, to timely detect and predict parotid gland shrinkage during/after (chemo)radiotherapy for oropharyngeal cancer. In order to maximize the potential benefits of adaptive re-planning for those patients who undergo significant parotid shrinkage, early evaluation of radiation-induced parotid gland modifications, possibly during the first 2 weeks of treatment [8, 27], is advised.

Anatomical change of parotid glands during radiotherapy may have dosimetric as well as clinical implications. The former is related to the higher dose received during treatment by the glands that show the larger variations [7, 8]; the latter to some extent that early anatomical modifications of parotids are associated with a reduction in salivary output, both acute and late [4, 26–28]. However, further investigation is underway to corroborate this point.

We found mean parotid shrinkages, ∆Vol_10fr_ and ∆Vol_post_, in satisfactory agreement with those reported by other investigators [7, 8]. Interestingly, we observed relatively similar values for both ADC and D_t_ across patients at baseline, suggesting that the glandular tissue is remarkably similar in diffusion coefficients in different patients, even if a small but statistically significant positive correlation was found between ADC and age (Additional file 1: Table S1 and Additional file 2: Figure S1). Such limited variability may have contributed to the lack of association between these parameters and parotid shrinkage, both during and after RT. Conversely, DCE-MRI features were highly different across patients at baseline, suggesting highly individual perfusion patterns. Moreover, the inverse relationship between BMI and DCE-MRI parameters (Additional file 1: Table S1 and Additional file 2: Figure S2) suggests a reduced proportional volume of vascular tissue in overweight patients. We also found that hypo-perfused parotids tend to show reduced ADC and D_t_ values, thus a reduced water molecule mobility (Additional file 1: Table S2). One may argue that this is indirectly related to the fact that the adipose tissue limits the mobility of water molecules as shown by Chang et al. [29], though no direct correlation was found between diffusion and BMI in our data. This suggests that the diffusion parameters, especially ADC and D_t_, are not just a simple expression of cellularity but seem to be dependent upon the characteristics of the glandular tissue (i.e. the proportion of glandular versus fatty components). To this end, we show that both diffusion and perfusion imaging may be useful to individually assess/monitor structural and functional properties of parotids.

Selected DCE-MRI parameters, such as K^trans^ and K_ep_, showed potential to detect parotid shrinkage both during and early after IMRT: patients with more vascularized parotid glands exhibited a greater volume reduction, possibly due to a higher tissue oxygenation and thus an increased radiosensitivity [5] (see also Figs. 3 and 4). On the other hand, Lee et al. [30] reported an inverse association between parotid shrinkage 3 months after RT for nasopharyngeal cancer and plasma volume derived from DCE-MRI. According to the Authors a higher plasma volume translates into a better perfusion thought it remains unclear how this would lead to an improved protection to radiation damage.

A previous study of Houweling et al. [21] also investigated the role of MRI to quantify the early radiation-induced modifications of the salivary glands in a population of patients with oropharyngeal carcinoma. The Authors documented a strong correlation between the increase in T2-weighted signal from baseline to 6 weeks after RT and the planned mean dose to parotid and submandibular glands. After treatment, they also found a decrease in k_ep_ and an increase in v_e_ which have been explained by a presumed rise in water content due to vascular edema. Similarly, we found an increase in ADC and D_t_, suggesting a larger extra-cellular/extra-vascular space allowing a higher tissue water mobility.

Initial BMI and patient weight were significantly correlated to ∆Vol_10fr_ consistent with a greater fatty component resulting in a smaller gland shrinkage, suggesting that in the first few weeks of treatment the glandular tissue is targeted preferentially over the adipose one [22]. The inverse relationship between parotid volume at baseline and ∆Vol_10fr_ likely reflects the strong positive correlation between the PG volume at baseline and both BMI and patient weight.

Parotid shrinkage at 2 weeks was found to be weakly related to both PG D_mean_ and V_30_, suggesting that the radiation-induced loss of acinar cells is a dose-dependent effect as previously shown [8, 23]. Accordingly, we found D_mean_ and V_30_ to be correlated to changes of ADC/D_t_ during the first 2 weeks of treatment that are presumed to reflect variations of cellular density (Additional file 1: Table S3). Moreover, at 2 weeks, the perfusion-free diffusion coefficient, D_t_, was found to be more predictive of PG shrinkage than ADC at either checkpoint (Table 4) suggesting a higher sensitivity in variations in cellular microstructure/depopulation than ADC [23] which is susceptible to the effects of both perfusion and diffusion [15]. The present data are also in agreement with the association between parotid shrinkage and parotid density reduction (acinar cell depopulation and relative increase in fibrofatty tissue) observed in CT-based studies [22].

Similarly to others [9], we could not find an association between parotid shrinkage after radiotherapy and both D_mean_ and V_30_, while a correlation was present after the first 2 weeks of treatment. There are multiple possible explanations for this change in predictive power of dose: progressive deterioration of the dose distribution throughout the course of treatment; delivery of a threshold dose above which parotid shrinkage is minimal; other confounding factors, such as weight loss or variability in parotid gland composition [7–9, 21, 27]. Conversely, earlier during treatment, shrinkage is likely to reflect more closely the depopulation of acinar cells, and thus, dose may play a more prominent and detectable role [8, 27]. Moreover, the limited variance in dose values and the small sample size may have contributed to mask an effect of dose on parotid shrinkage after treatment.

It is unclear why ∆Vol_post_ was related to in-treatment *f* values and changes in *f*, as no significant association was observed between initial perfusion-related diffusion coefficients (D*, f and D* × *f*) and any DCE-MRI perfusion parameters. However, contradictory results on the ability of IVIM-DWI in assessing tissue perfusion have been reported in the literature to date [17]. This may reflect either the presence of confounding sources of incoherent motion in biological tissues and/or the difficulty to obtain an optimal image quality with minimal motion and susceptibility artifacts, particularly in patients with head and neck cancer [15, 17].

The study has some limitations. We extracted the median value of the signal from all the voxels within the entire gland as opposed to the punctual ‘local’ signal from discrete sub-regions of the PG. We also did not investigate the ability of MRI to detect a different local effect of radiation dose, as suggested by others [21]; this may help to identify sub-regions with different radiosensitivity within the gland [31]. On the other hand, we believe that our approach allows the extraction of data less susceptible to motion artifacts and image co-registration issues and ultimately more consistent. Future investigations from this on-going study will include both patient-reported xerostomia as an endpoint as soon as clinical data mature and a multivariable model on selected quantitative image analyses, in addition to patient- and treatment-related covariates.

## Conclusions

Both IVIM-DWI and DCE-MRI can help to detect early (during treatment) as well as late (after treatment completion) PG shrinkage. Moreover, they shed light upon the complex correlation between the volumetric change of PG and patient−/treatment-related variables by assessing individual microcapillary perfusion and tissue diffusivity.

## Additional files

Additional file 1: **Table S1.** Spearman’s Rho values between DCE-MRI/IVIM-DWI at baseline and anthropometric variables at baseline. **Table S2.** Spearman’s Rho values between DCE-MRI and IVIM-DWI parameters at baseline. **Table S3.** Spearman’s Rho values among changes of IVIM-DWI parameters during treatment and dosimetric variables. (DOCX 22 kb)

Additional file 2: **Figure S1.** Scatter plots of IVIM-DWI parameters versus Age and BMI. **Figure S2.** Scatter plots of DCE-MRI parameters versus Age and BMI. (ZIP 212 kb)

## Abbreviations

ADC: Apparent diffusion coefficient
BMI: Body mass index
D* × *f*: Product of D* by *f*
D*: Perfusion-related diffusion coefficient
D_mean_: Planned mean dose to the parotid gland
D_t_: Tissue diffusion coefficient
EES: Extravascular extracellular space
*f*(%): Perfusion fraction
IAUGC: Initial area under gadolinium concentration curve
IMRT: Intensity modulated radiotherapy
K_ep_: Transfer constant between EES and plasma
K^trans^: Transfer constant between plasma and EES
PG: Parotid gland
V_30_(%): Percentage of parotid volume receiving a dose ≥30 Gy
v_e_: Fractional volume of EES
∆Vol_10fr_: Parotid shrinkage(%) at the 10th fraction
∆ADC: ADC variation (%) relative to the pretreatment value (analogously for the other imaging variables)
∆Vol_post_: Parotid shrinkage(%) 8 weeks after RT
