# Supplementary material for: Radiation-induced parotid changes in oropharyngeal cancer patients: the role of early functional imaging and patient−/treatment-related factors
Source: Radiat Oncol. 2018 Oct 1;13:189. doi: 10.1186/s13014-018-1137-4 (PMC6167883; doi:10.1186/s13014-018-1137-4)
Supplement: Supplementary file 1 — Table S1. Spearman’s Rho values between DCE-MRI/IVIM-DWI at baseline and anthropometric variables at baseline. Table S2. Spearman’s Rho values between DCE-MRI and IVIM-DWI parameters at baseline. Table S3. Spearman’s Rho values among changes of IVIM-DWI parameters during treatment and dosimetric variables. (DOCX 22 kb) [file 13014_2018_1137_MOESM1_ESM.docx]

**Table S1** Spearman's Rho values between DCE-MRI/IVIM-DWI at baseline and anthropometric variables at baseline.

| Parameter  at baseline | Age | | BMI | | PG volume | |
| --- | --- | --- | --- | --- | --- | --- |
|  | Rho | P | Rho | p | Rho | P |
| K^trans^ | 0.129 | 0.266 | -0.353 | **0.002** | -0.057 | 0.623 |
| K_ep_ | 0.038 | 0.747 | -0.205 | 0.076 | -0.013 | 0.913 |
| v_e_ | -0.084 | 0.477 | -0.246 | **0.035** | -0.263 | 0.023 |
| IAUGC | 0.065 | 0.576 | -0.374 | **0.001** | -0.179 | 0.121 |
| ADC | 0.260 | **0.020** | 0.06 | 0.58 | 0.075 | 0.510 |
| D_t_ | 0.084 | 0.461 | 0.01 | 0.97 | 0.156 | 0.170 |
| *f* | 0.268 | **0.016** | 0.16 | 0.16 | -0.126 | 0.270 |
| D* | -0.081 | 0.476 | 0.026 | 0.82 | -0.052 | 0.646 |
| D*×*f* | -0.021 | 0.852 | 0.075 | 0.51 | -0.152 | 0.179 |

Statistically significant p-values are bold.

**Table S2** Spearman's Rho values between DCE-MRI and IVIM-DWI parameters at baseline.

| Parameter | K^trans^ | | K_ep_ | | v_e_ | | IAUGC | |
| --- | --- | --- | --- | --- | --- | --- | --- | --- |
|  | Rho | p | Rho | P | Rho | P | Rho | p |
| ADC | 0.082 | 0.481 | 0.026 | 0.824 | 0.018 | 0.876 | 0.063 | 0.589 |
| D_t_ | 0.242 | **0.035** | 0.103 | 0.377 | 0.145 | 0.217 | 0.230 | **0.045** |
| *f* | -0.085 | 0.465 | 0.001 | 0.995 | -0.098 | 0.405 | -0.086 | 0.461 |
| D* | -0.005 | 0.967 | 0.032 | 0.787 | -0.106 | 0.368 | -0.089 | 0.447 |
| D*×*f* | -0.084 | 0.472 | 0.046 | 0.696 | -0.195 | 0.095 | -0.186 | 0.107 |

Statistically significant p-values are bold.

**Table S3** Spearman's Rho values among changes of IVIM-DWI parameters during treatment and dosimetric variables.

| Parameter | ΔADC | | ΔD*_t_* | | Δ*f* | | ΔD* | | ΔD*×*f* | |
| --- | --- | --- | --- | --- | --- | --- | --- | --- | --- | --- |
|  | Rho | P | Rho | p | Rho | P | Rho | P | Rho | p |
| D_mean_(Gy) | 0.356 | **0.001** | 0.281 | **0.011** | 0.238 | **0.033** | -0.231 | **0.040** | -0.134 | 0.236 |
| V_30_(%) | 0.404 | **0.000** | 0.349 | **0.001** | 0.226 | **0.043** | -0.159 | 0.160 | -0.072 | 0.527 |

Statistically significant p-values are **bold**.

**Abbreviations**

PG, parotid gland; BMI, body mass index; D_mean_, planned mean dose to the parotid gland; V_30_(%), percentage of parotid volume receiving a dose ≥ 30 Gy; EES, extravascular extracellular space; K^trans^, transfer constant between plasma and EES; K_ep_, transfer constant between EES and plasma; v_e_, fractional volume of EES; IAUGC, initial area under gadolinium concentration curve; ADC, apparent diffusion coefficient; D_t_, tissue diffusion coefficient; D*, perfusion-related diffusion coefficient; *f*(%), perfusion fraction; D*× *f*, product of D* by *f*; ΔADC, ADC variation (%) relative to the pretreatment value (analogously for the other imaging variables).
