# Supplementary figures and images for: Radiation-induced parotid changes in oropharyngeal cancer patients: the role of early functional imaging and patient−/treatment-related factors
Source: Radiat Oncol. 2018 Oct 1;13:189. doi: 10.1186/s13014-018-1137-4 (PMC6167883; doi:10.1186/s13014-018-1137-4)

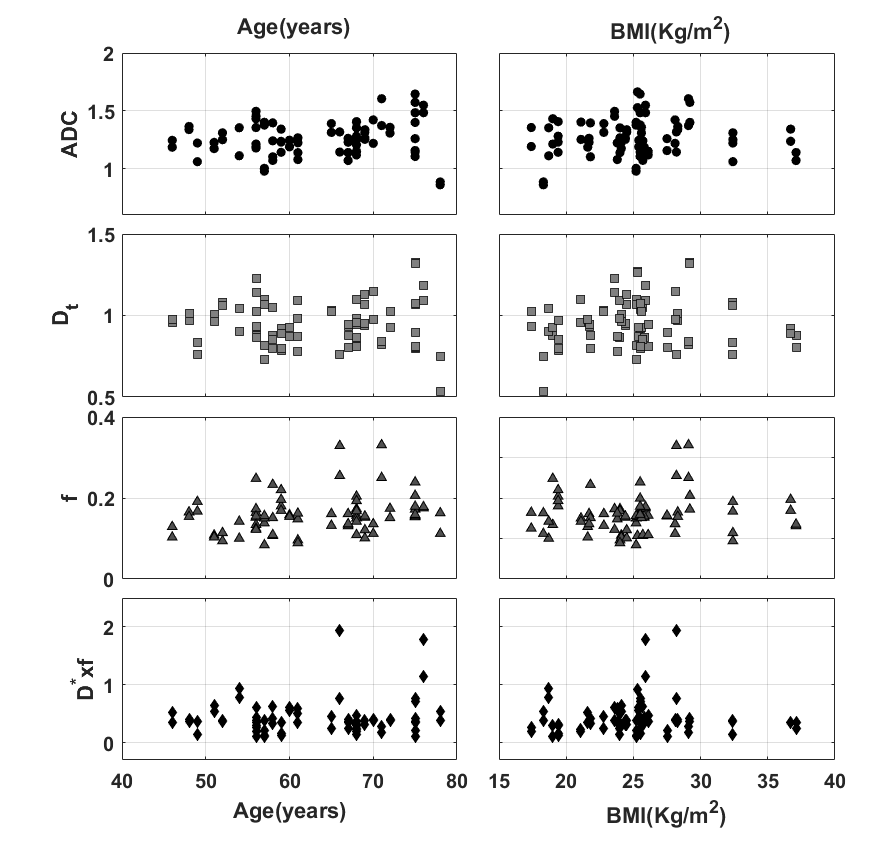

Supplement: Supplementary file 2 — Figure S1. Scatter plots of IVIM-DWI parameters versus Age and BMI. Figure S2. Scatter plots of DCE-MRI parameters versus Age and BMI. (ZIP 212 kb) [file 13014_2018_1137_MOESM2_ESM.zip › SupplementaryFigure2.tif]

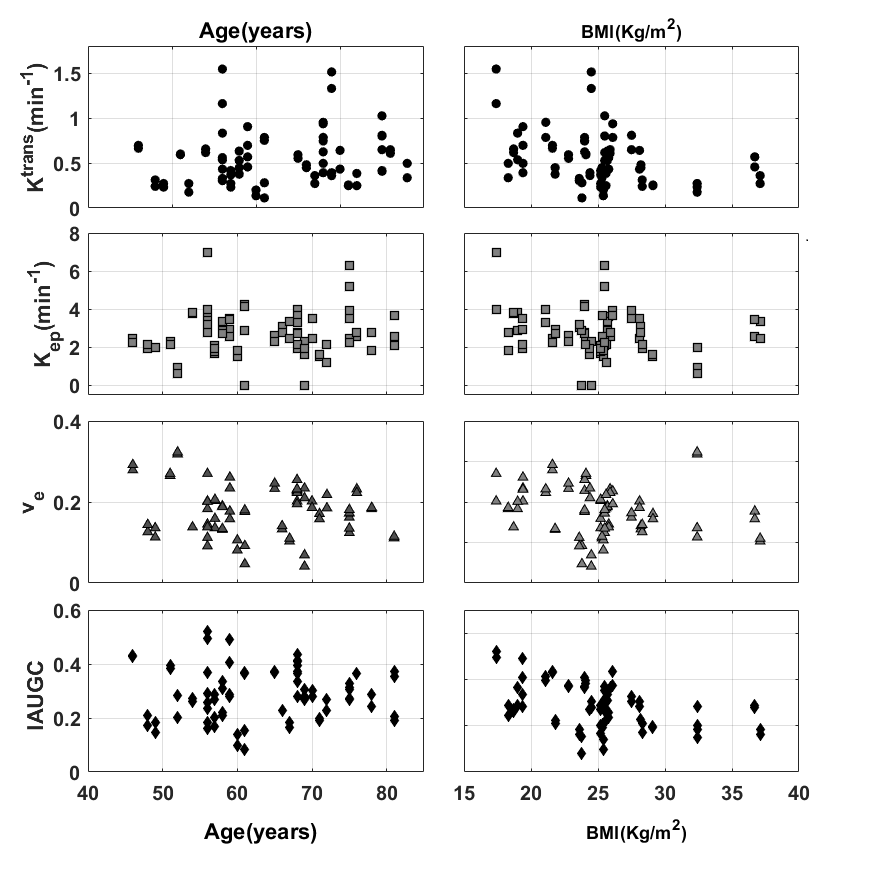

Supplement: Supplementary file 2 — Figure S1. Scatter plots of IVIM-DWI parameters versus Age and BMI. Figure S2. Scatter plots of DCE-MRI parameters versus Age and BMI. (ZIP 212 kb) [file 13014_2018_1137_MOESM2_ESM.zip › SupplementaryFigure1.tif]
